# Reflections Upon 55 Years of the First Human Heart Transplant in Brazil

**DOI:** 10.21470/1678-9741-2023-0208

**Published:** 2024-02-26

**Authors:** Noedir Antonio Groppo Stolf

**Affiliations:** 1 Cardiovascular Surgery Department, Instituto do Coração, Universidade de São Paulo, São Paulo, São Paulo, Brazil

The first human heart transplant in Brazil was performed by Dr. Zerbini and an associate in the dawn of May 26^th^, 1968. Cardiac transplantation in dogs had been done previously by Dr. Euclydes Marques and a group of medical students from 1962 through 1964. The experience with 30 transplanted dogs was presented at the XXIII Congresso Brasileiro de Cardiologia (Figure 1)^[1]^. A proposal of human cardiac transplantation was made twice to the Chairman of the Thoracic Surgery Department and in a meeting of the Department of Cardiology at Hospital das Clínicas, Faculdade de Medicina, Universidade de São Paulo. On both occasions, it was considered premature.

**Fig. 1:**
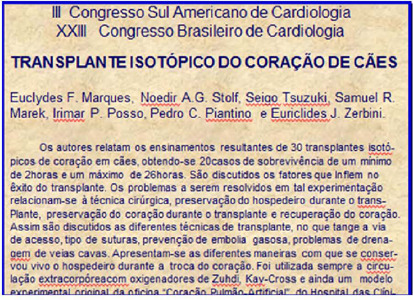
Abstract of the paper presented at the XXVIII Congresso Brasileiro de Cardiologia.

On December 3^rd^, 1967, the first interhuman heart transplant was done at the Groote Schuur Hospital, University of Cape Town (South Africa) by a team led by Dr. Christian N. Barnard (Figure 2). Immediately, the extraordinary feat spread in local and international media (Figure 3). The recipient survived 18 days and died from pneumonia with a missed diagnosis of rejection.

**Fig. 2:**
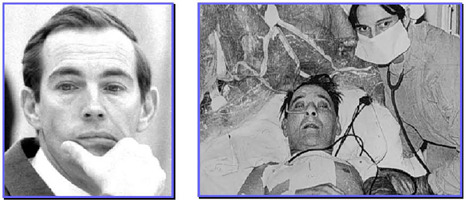
Dr. Barnard (left) and Washkansky, the first recipient, (right).

**Fig. 3:**
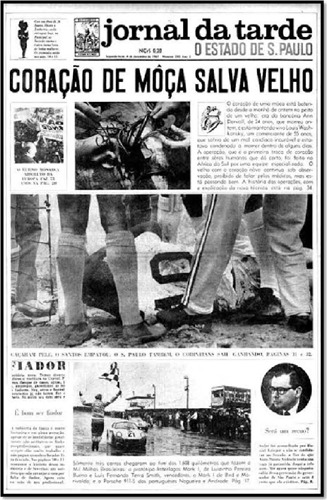
Headline of Jornal da Tarde (São Paulo newspaper) of December 4^th^, 1967.

After the notice of Barnard’s achievement, Dr. Euryclides de Jesus Zerbini decided to prepare for cardiac transplantation on December 4^th^, 1967. A team of surgeons, cardiologists, and a hematologist, who served as an immunologist, started to discuss in detail selection of recipient and donor, heart preservation, immunosuppression, and other issues in many meetings.

The first recipient was a patient with dilated cardiomyopathy, and the donor was a patient with skull trauma, loss of brain tissue through the wound, and tracheotomy. Electroencephalogram was performed, with physical and light stimulation to confirm brain death diagnosis made by an independent neurologist. A canula was inserted in the innominate artery and connected to a cardiopulmonary bypass circuit. Ventilation was discontinued and it was waited until heartbeat stopped. Then a selective perfusion of the heart was started, and the donor heart was harvested. Meanwhile, the recipient was prepared, in an adjacent operating room, and the heart was resected. The perfused donor heart was transported in a basin and prepared in the recipient operating room. Cardiac transplantation was done by the so-called conventional technique. The recipient by the name of João Ferreira became in the media nationwide known as João “boiadeiro” (Figures 4 and 5). He survived 28 days and died from refractory acute rejection (Figure 5). Two other patients were transplanted by Dr. Zerbini in 1968 and 1969; one (the second) survived more than one year (Figure 6), and the other (the third) survived for 60 days.

**Fig. 4:**
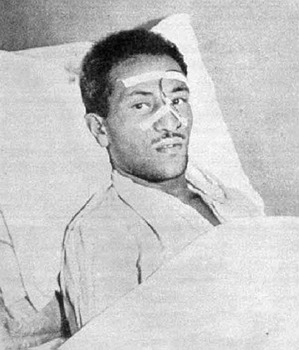
João “boiadeiro”, the first recipient of heart transplantation in Brazil.

**Fig. 5:**
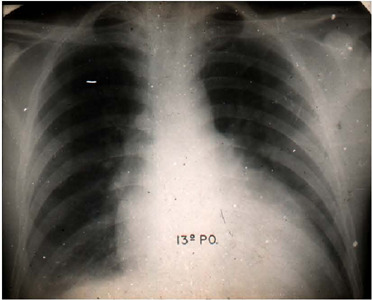
Radiography of João “boiadeiro” at the 13^th^ postoperative day.

**Fig. 6:**
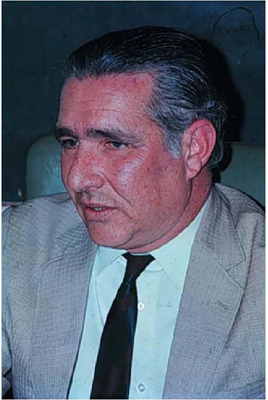
Picture of the second transplanted patient around one year after transplantation.

Heart transplant in Brazil resumed in the second semester of 1984, with the transplantation performed by Nesralla and associates at Instituto de Cardiologia do Rio Grande do Sul.

According to the Registro Brasileiro de Transplantes (RBT) of the Associação Brasileira de Transplante de Órgãos (or ABTO) in its 2022 report^[3]^, notification of potential donors is increasing but the actual donation remains very low (26.9%). Family refusal of donation increased (47%) as well as medical contraindication (17%). These increases reflect some circumstances related to the Coronavirus disease 2019 pandemic as well as the inadequate care of potential donors. The number of cardiac transplant per million persons was the same in the last 10 years[^1,7^].

The number of transplants per year from 2015 to 2022 was 353, 357, 380, 358, 380, 308, 334, and 359, respectively, according to the RBT. São Paulo state was the one with more transplants in 2022 (133) followed by Minas Gerais (64), Rio de Janeiro (34), Brasília (32), and Pernambuco (32). There are 76 services of cardiac transplantation through the country, there were 340 patients on the waiting list for transplantation at the end of 2022, and there are 11 states with no patient on the waiting list. During the year of 2022, 432 patients entered the waiting list, and 105 died while on the waiting list (pediatric and adult patients).

Considering the increasing incidence of heart failure in most countries and that the mortality of this condition is higher than cancer mortality, for instance, it is a matter of concern that we don’t have an increase in heart transplantation in the last 10 years. Donor shortage is an important issue to be faced by public authorities and societies with campaign and media, but it is not the only one. An adequate care of the potential donor is very important to increase the number of donors. Another important aspect is the payment to the hospitals and heart transplantation teams. The transplantation affects the routine agenda of cardiovascular surgeons as other urgent or emergency procedures but requires more resources to harvest and transplant the heart.

In conclusion, the annual number of heart transplants in Brazil is very low, one third of what is expected for the Brazilian population. Furthermore, this number is not increasing in the last 10 years. It is urgent that Government, Cardiovascular Surgery Societies, and Transplantation Societies implement new policies and strength the present ones to face this problem.

## References

[1] Marques EF, Stolf NAG, Tsuzuki S, Marek S, Posso I, Piantino PC (1962). Transplante isotópico do coração em
cães.

[2] Stolf NAG. (2017). History of heart transplantation: a hard and glorious
journey. Braz J Cardiovasc Surg.

[3] RBT - Registro Brasileiro de Transplantes, ABTO – Associação Brasileira de Transplante. (2022). Dimensionamento dos transplantes no Brasil e em cada estado (2015-2022)
[Internet].

